# Effects of particulate matter on hospital admissions for respiratory diseases: an ecological study based on 12.5 years of time series data in Shanghai

**DOI:** 10.1186/s12940-021-00828-6

**Published:** 2022-01-13

**Authors:** Wenjia Peng, Hao Li, Li Peng, Ying Wang, Weibing Wang

**Affiliations:** 1 Department of Epidemiology, School of Public Health, Fudan University, Shanghai, 200032 China; 2 Department of Epidemiology, Shanghai Key Laboratory of Meteorology and Health, Shanghai, 200032 China; 3 Key Laboratory of Health Technology Assessment, National Health and Family Planning Commission of the People’s Republic of China, Fudan University, Shanghai, China; 4 Department of Social Medicine, School of Public Health, Fudan University, Shanghai, 200032 China; 5 IRDR-ICoE on Risk Interconnectivity and Governance on Weather/Climate Extremes Impact and Public Health, Fudan University, Shanghai, 200032 China

**Keywords:** Particulate matter, Respiratory diseases, Hospital admission, Time series

## Abstract

**Background:**

Previous epidemiological studies on the association between short-term exposure to particulate matter (PM) with hospital admission in major cities in China were limited to shorter study periods or a single hospital. The aim of this ecological study based on a 12.5-year time series was to investigate the association of short-term exposure to PM with aerodynamic diameter ≤ 2.5 μm (PM_2.5_) and aerodynamic diameter ≤ 10 μm (PM_10_) with hospital admissions for respiratory diseases.

**Methods:**

Daily hospital admissions data were from the Shanghai Medical Insurance System for the period January 1, 2008 to July 31, 2020. We estimated the percentage change with its 95% confidence interval (CI) for each 10 μg/m^3^ increase in the level of PM_2.5_ and PM_10_ after adjustment for calendar time, day of the week, public holidays, and meteorological factors applying a generalized additive model with a quasi-Poisson distribution.

**Results:**

There were 1,960,361 hospital admissions for respiratory diseases in Shanghai during the study period. A 10 μg/m^3^ increase in the level of each class of PM was associated with increased total respiratory diseases when the lag time was 0 day (PM_2.5_: 0.755%; 95% CI: 0.422, 1.089%; PM_10_: 0.250%; 95% CI: 0.042, 0.459%). The PM_2.5_ and PM_10_ levels also had positive associations with admissions for COPD, asthma, and pneumonia. Stratified analyses demonstrated stronger effects in patients more than 45 years old and during the cold season. Total respiratory diseases increased linearly with PM concentration from 0 to 100 μg/m^3^, and increased more slowly at higher PM concentrations.

**Conclusions:**

This time-series study suggests that short-term exposure to PM increased the risk for hospital admission for respiratory diseases, even at low concentrations. These findings suggest that reducing atmospheric PM concentrations may reduce hospital admissions for respiratory diseases.

**Supplementary Information:**

The online version contains supplementary material available at 10.1186/s12940-021-00828-6.

## Background

Respiratory diseases, including chronic obstructive pulmonary disease (COPD), asthma, interstitial lung disease, and pulmonary sarcoidosis, are major public health problems that impose a huge disease burden on society [1]. The disability-adjusted life years (DALYs) of respiratory diseases increased from 97.2 to 112.3 million a year from 1990 to 2017 [2]. There are two well-known risk factors associated with respiratory diseases: behavioral factors (e.g. smoking) and environmental factors (e.g. air pollution) [2].

China is now faced with severe air pollution due to rapid urbanization and industrialization, especially in the more populous areas. According to the global burden of disease study 2017 [3], 1.24 million deaths were attributable to air pollution exposure, and air pollution resulted in 40.0 and 35.6% of DALYs for COPD and lower respiratory infections in China, respectively. To cope with the severe air pollution problem, the Chinese government has implemented several policies and regulations to improve the air quality and issued the Chinese National Ambient Air Quality Standard (GB3095–2012) in 2012, which limited the annual and 24-h mean concentrations of the major pollutants.

The most common pollutants are particulate matter (PM) with an aerodynamic diameter less than or equal to 2.5 μm (PM_2.5_) and with an aerodynamic diameter less than or equal to 10 μm (PM_10_). Longtime exposure to PM can cause multiple health problems that reduce human longevity [4]. Although numerous ecological studies have investigated the association between PM and outpatient or inpatient admissions of respiratory diseases, most of them were usually conducted for a relatively shorter study period [5–8] or a single hospital [7, 9] with a limited number of outcomes. Moreover, some studies [5, 8, 10] on the association of PM with respiratory diseases have produced inconsistent results.

Shanghai is the most populous megacity with a permanent population of 24.8 million in the seventh national census. It is also one of the heaviest polluted cities in China. The present study used time-series data for 12.5 years from all the medical insurance designated hospitals from Shanghai Healthcare Security Database for Urban Employees and Urban and Rural Residents. The database covers more than 95% of the adult population in Shanghai according to statistics released by the National Healthcare Security Administration, resulting in having a large number of hospitalizations and therefore providing good statistical power to examine the respiratory disease subcategories. We have investigated the association of short-term exposure to PM_2.5_ and PM_10_ with total respiratory diseases and subcategories (COPD, asthma, and pneumonia) in Shanghai.

## Methods

### Respiratory disease data

Daily hospital admissions data were from the Shanghai Medical Insurance System for the period January 1, 2008 to July 31, 2020. The clinical diagnostic criteria for respiratory diseases were from the International Classification of Diseases, 10th Revision as J00 to J99. This included COPD (J40–J44), asthma (J45), and pneumonia (J12–J18). Patients’ basic information included gender and age (< 45 years, 45–64 years, 65–74 years, and ≥ 75 years). Prior to data collection, this study was approved by the Ethics Committee of the School of Public Health, Fudan University.

### Air pollutants and meteorological data

**Fig. 1:**
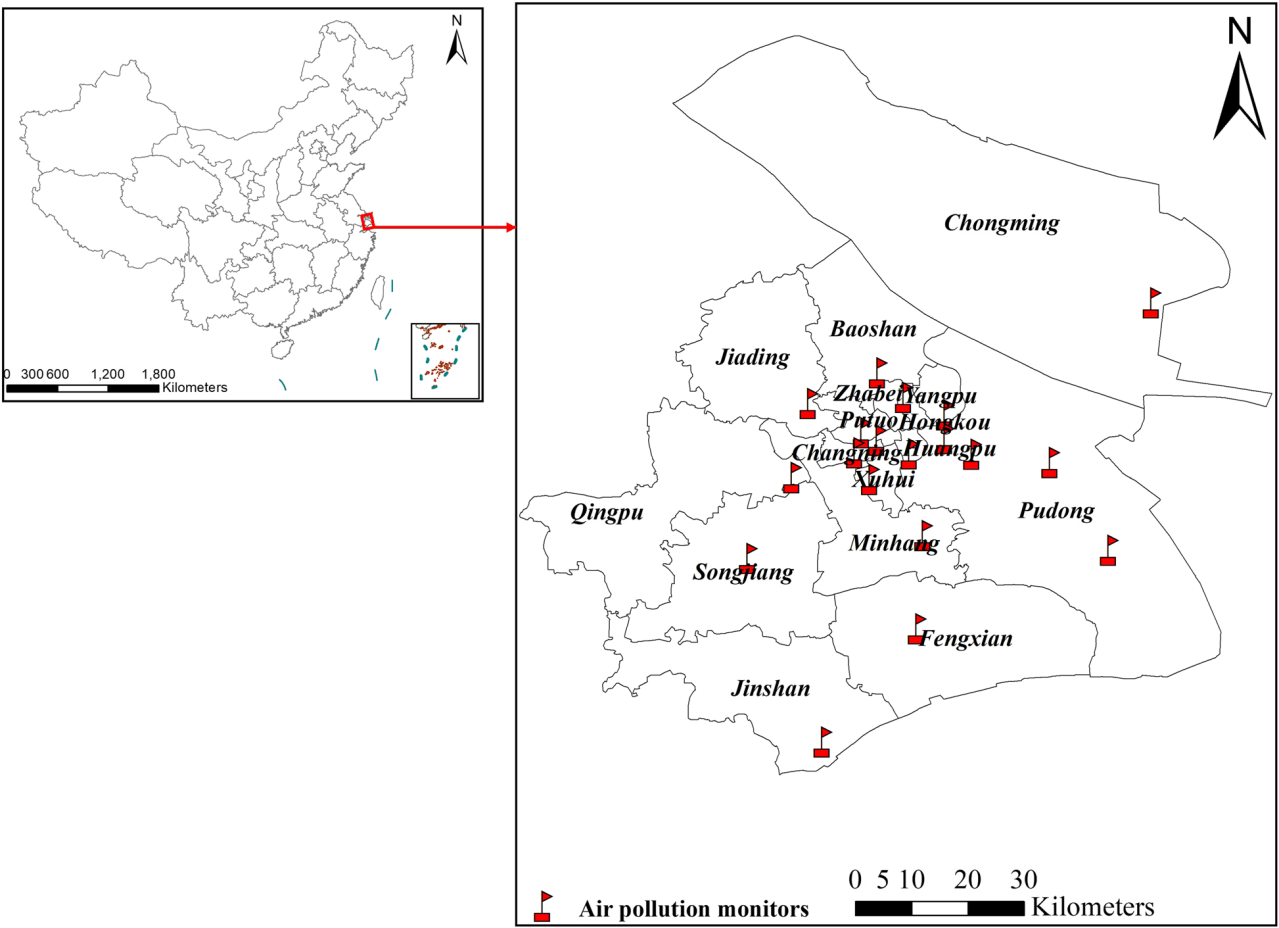
Locations of air pollution monitors in Shanghai

We collected six of the most common air pollutants from the Shanghai Municipal Bureau of Ecological Environment (https://sthj.sh.gov.cn/), namely PM_2.5_, PM_10_, nitrogen dioxide (NO_2_), sulfur dioxide (SO_2_), ozone (O_3_), and carbon monoxide (CO). Of these, the daily concentrations of PM_10_, NO_2_, and SO_2_ were available from January 1, 2008 to July 31, 2020. While, daily PM_2.5_, O_3_, and CO concentrations were only available after the establishment of the ground monitoring network after January 1, 2013.There are 19 environmental monitoring stations in Shanghai (Fig. 1). The daily concentration of air pollutants was simply an arithmetic mean measure across all the monitoring stations, as in most time-series studies. Meteorological data (mean temperature and relative humidity) in Shanghai were from the National Meteorological Information Center (http://data.cma.cn/).

### Statistical analysis

A generalized additive model (GAM) [11] with a quasi-Poisson distribution was adopted to analyze the impact of PM_2.5_ and PM_10_ on daily hospital admissions of respiratory diseases. The effect of different time lags was examined including six single-day lags: (*i*) lag 0, the present day; (*ii*) lag 1, the previous day; (*iii*) lag 2, the day before lag 1; (*iv*) lag 3, the day before lag 2; (*v*) lag 4, the day before lag 3; (*vi*) lag 5, the day before lag 4, and three moving average exposure lags: (*i*) lag 01, the 2-day moving average of the present and previous day; (*ii*) lag 02, the 3-day moving average of the present and previous 2 days; (*iii*) lag 03, the 4-day moving average of the present and previous 3 days.

Based on previous studies [12, 13], the following covariates were used: (*i*) a natural spline function of calendar time with 7 degrees of freedom per year (to exclude unmeasured time trends); (*ii*) a natural spline function with 6 degrees of freedom for present-day mean temperature and 3 degrees of freedom for present-day relative humidity (to control for the nonlinear confounding effects of weather conditions); and (*iii*) indicator variables for day of the week (DOW) and public holidays (PH). Therefore, the main model was:

$$Log\left(E\left({Y}_t\right)\right)=\alpha +\beta \times {Z}_t+ ns\left( time, df=7/ year\right)+ ns\left( temperature, df=6\right)+ ns\left( relative\ humidity, df=3\right)+ as. factor(DOW)+ as. factor(PH)$$

where *E*(*Y*_*t*_) is the estimated daily hospital admissions for respiratory diseases, *Z*_*t*_ is the PM concentration on day t, *β* is the regression coefficient for *Z*_*t*_, and *α* is the intercept. All results were presented as percentage change with a 95% confidence interval (CI) for each 10 μg/m^3^ increase of PM. Percentage change was calculated using the following formula [14]:

$$Percentage\ Change=\left[\exp \left(\beta \times 10\right)-1\right]\times 100$$

where *β* is the regression coefficient of PM from the GAM model.

Effect modification by individual characteristics was investigated using stratified analyses. The subgroup variables included gender (male and female), age (< 45, 45-, 65- and 75- years), and season (cold: November, December, January, February, March and warm: April, May, June, July, August, September, October). The subgroup differences were tested using a Z statistic [15]:

$$Z=\left({\beta}_1-{\beta}_2\right)/\sqrt{SE_1^2-{SE}_2^2}$$

where *β*_1_and *β*_2_ are the effect estimates of the two subgroups, *SE*_1_ and *SE*_2_ are their corresponding standard errors.

A cubic spline smoothing method was used to evaluate the exposure-response relationship between PM_2.5_ and PM_10_ concentrations and hospital admissions for respiratory diseases.

The attributable fraction (AF) and attributable number (AN) of hospital admissions due to PM exposure were estimated using the following formula based on a previous study [14]:

$$AF=1-\frac{1}{\exp \left[\beta \times \left(C-{C}_0\right)\right]}$$

$$AN= AF\times N$$

where *AF* is the daily attributable fraction, *AN* is the daily attributable number of hospital admissions due to PM, *N* is the daily hospital admissions, *β* is the regression coefficient (from the main model above), *C* is the daily PM concentration, and *C*_0_ is the reference PM concentration. The reference concentrations were from the air quality guidelines of the WHO: 24-h mean: 15 μg/m^3^ for PM_2.5_ and 45 μg/m^3^ for PM_10_. The total attributable number was estimated by summing daily AN.

We also performed three sensitivity analyses to determine the robustness of the results. First, a two-pollutant model (PM_2.5_, PM_10_ with O_3_, SO_2_, NO_2_, CO) was fitted. Second, we changed the degrees of freedom for the calendar time from 5 to 9 per year. Third, we excluded the data from 2020 due to the coronavirus disease 2019 pandemic.

All data processing and statistical analyses were conducted using R software (version 3.6.1).

## Results

### Hospital admissions for respiratory diseases and meteorological variables

There were 1,960,361 hospital admissions for total respiratory diseases (665,541 for COPD, 455,718 for pneumonia, and 33,329 for asthma) in Shanghai from January 1, 2008 to July 31, 2020 included in our analysis (Table 1). Males accounted for 55.69% of the patients and 49.85% of the patients were older than 75 years old.

**Table 1:** Characteristics of hospital admissions for respiratory diseases in Shanghai, China from January 1, 2008 to July 31, 2020

Characteristics	n (%)
Admissions of total respiratory diseases	1,960,361 (100.00)
COPD	665,541 (33.95)
Asthma	33,329 (1.70)
Pneumonia	455,718 (23.25)
Gender	
Male	1,091,645 (55.69)
Female	868,714 (44.31)
Missing	2 (0.00)
Age groups, years	
< 45	201,952 (10.30)
45–	410,273 (20.93)
65–	370,846 (18.92)
75–	977,289 (49.85)
Missing	1 (0.00)
Season	
Cold	885,737 (45.18)
Warm	1,074,624 (54.82)

Analysis of the air pollutants indicated the daily mean concentrations were 43.71 μg/m^3^ for PM_2.5_, 66.12 μg/m^3^ for PM_10_, 45.94 μg/m^3^ for NO_2_, 21.08 μg/m^3^ for SO_2_, 98.59 μg/m^3^ for O_3_, and 0.72 mg/m^3^ for CO (Table 2). Our analysis of the annual average concentrations indicated decreasing trends for PM_2.5_ and PM_10_ from 2013 to 2020 (Table S I).

**Table 2:** Summary statistics of air pollutants and meteorological variables in Shanghai, China from January 1, 2008 to July 31, 2020

Variables	Mean ± SD	Minimum	P25	Median	P75	Maximum
Air pollutants						
PM_2.5_, μg/m^3 *^	43.71 ± 31.18	5.00	22.00	36.00	56.00	255.00
PM_10_, μg/m^3^	66.12 ± 44.07	6.00	37.28	54.00	82.00	599.29
NO_2_, μg/m^3^	45.94 ± 20.31	5.00	31.00	43.00	57.00	141.65
SO_2_, μg/m^3^	21.08 ± 17.39	3.00	9.00	15.00	26.83	146.78
O_3_, μg/m^3 *^	98.59 ± 41.78	10.00	69.00	92.00	120.00	269.00
CO, mg/m^3 *^	0.72 ± 0.26	0.30	0.52	0.68	0.80	2.28
Meteorological factors						
Temperature, °C	17.33 ± 8.86	−6.20	9.50	18.30	24.60	35.70
Relative humidity, %	71.50 ± 12.77	23.00	63.00	72.00	81.00	100.00

*Note*: * 2013.1.1–2020.7.31, *P25* 25th percentile, *P75* 75th percentile, *SD* standard deviation

### Correlation between air pollutants and meteorological variables

We calculated Spearman correlation coefficients to examine the relationships of air pollution variables and meteorological factors (Table S II). The results indicated that daily PM_2.5_ and PM_10_ concentrations had positive correlations with NO_2_ (PM_2.5_: r_s_ = 0.721, *P* < 0.001; PM_10_: r_s_ = 0.685, P < 0.001), SO_2_ (PM_2.5_: r_s_ = 0.729, P < 0.001; PM 10: r_s_ = 0.655, P < 0.001), and CO (PM_2.5_: r_s_ = 0.879, P < 0.001; PM_10_: r_s_ = 0.759, P < 0.001). There were inverse correlations of PM_2.5_ and PM_10_ concentrations with temperature (PM_2.5_: r_s_ = − 0.311, P < 0.001; PM_10_: r_s_ = − 0.229, P < 0.001) and relative humidity (PM_2.5_: r_s_ = − 0.142, P < 0.001; PM_10_: r_s_ = − 0.365, P < 0.001).

### Effect estimates between PM and respiratory diseases

**Fig. 2:**
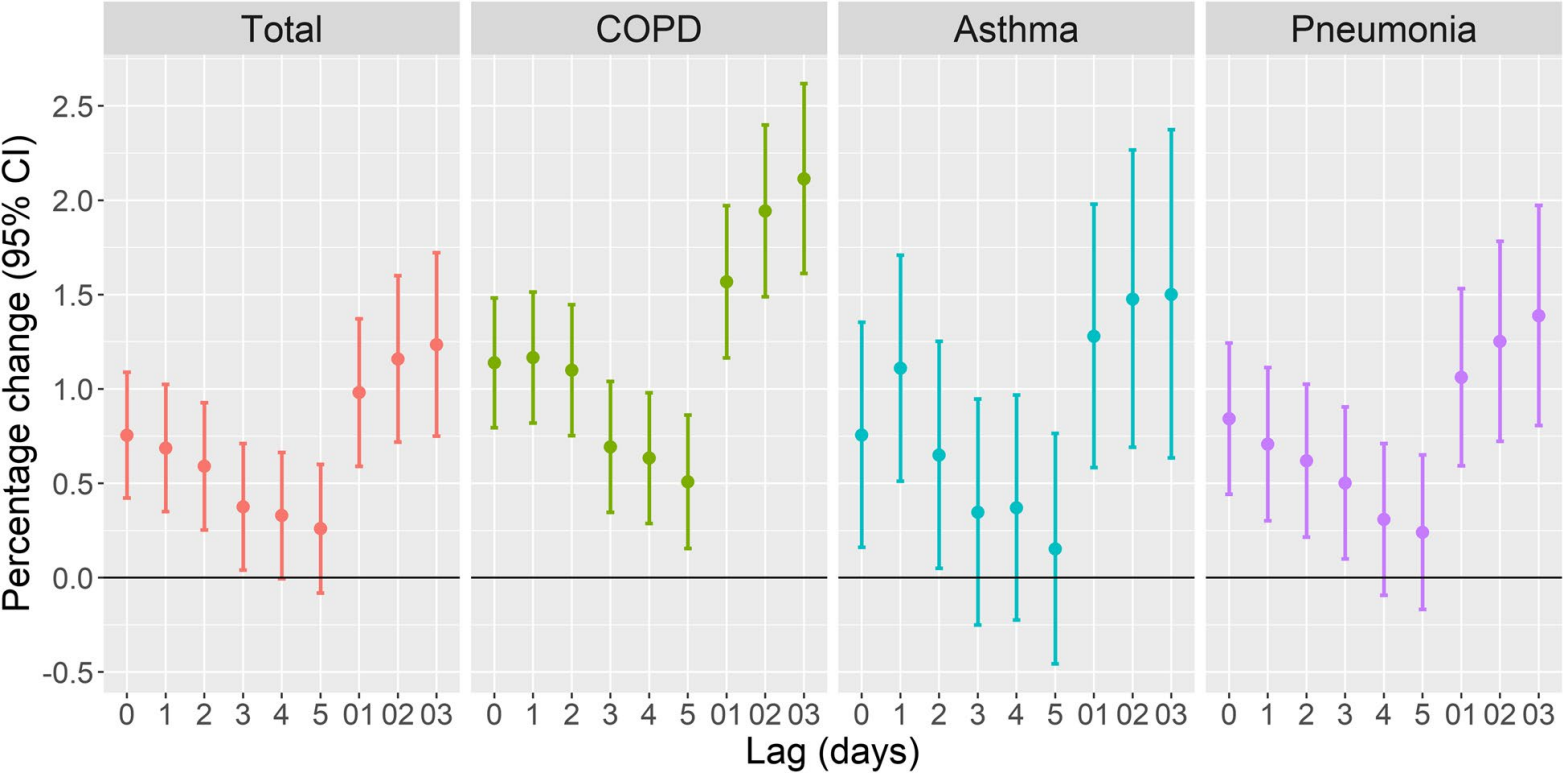
Percentage change (95% CI) in hospital admissions for respiratory diseases for each 10 μg/m^3^ increase in the level of PM_2.5_ in Shanghai from 2013 to 2020 and effect of lag time. All models were adjusted for public holidays, day of the week, and calendar day

**Fig. 3:**
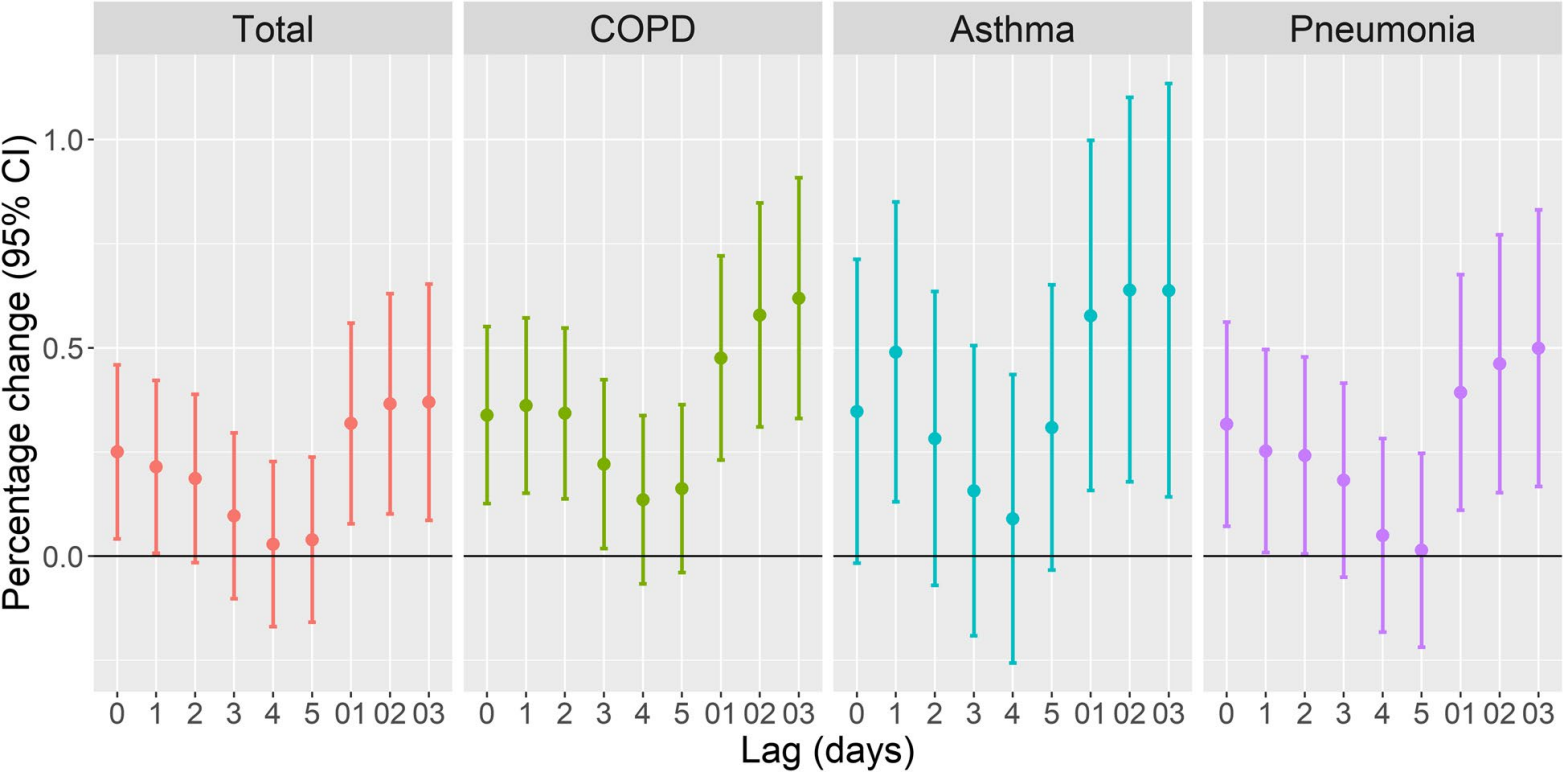
Percentage change (95% CI) in hospital admissions for respiratory diseases for each 10 μg/m^3^ increase in the level of PM_10_ in Shanghai from 2008 to 2020 and effect of lag time. All models were adjusted for public holidays, day of the week, and calendar day

We examined the effects of PM_2.5_ and PM_10_ on hospital admissions for respiratory diseases using different lag times (Figs. 2 and 3). Overall, a 10 μg/m^3^ increase of each PM was associated with increased total respiratory diseases at lag 0 (PM_2.5_: 0.755%; 95% CI: 0.422, 1.089%; PM_10_: 0.250%; 95% CI: 0.042, 0.459%). These associations decreased as the lag time increased to lag 5, but were increasingly greater for moving average exposure lags of lag01, lag02, and lag03. Analysis of each cause-specific respiratory disease indicated similar trends as total respiratory diseases. The percentage change seemed to be greater for COPD and asthma at lag 1. PM_2.5_ had a greater effect than PM_10_ for all lag times.

### Stratified analyses by gender, age, and season

Stratification by gender indicated the associations of PM_2.5_ and PM_10_ were similar in males and females (Table 3). Stratification by age indicated the associations of PM_2.5_ with total respiratory diseases, asthma, and pneumonia were more pronounced in patients more than 45 years old. For patients younger than 45 years, there were no significant associations of PM with total respiratory diseases or with individual diseases, except for PM_2.5_ and COPD. Stratification by season indicated the associations were more evident during the cold season, and that there were no significant associations of PM with total respiratory diseases or individual diseases during the warm season. We also analyzed these data using 9 different lag times (Figs. S I – Figs. S VI).

**Table 3:** Percentage change with 95% confidence interval for hospital admissions of respiratory diseases for a 10 μg/m3 increase in particulate matter, by gender, age groups, and season

Respiratory diseases	PM_2.5_		PM_10_	
	Percentage change (95%CI)	*P* value	Percentage change (95%CI)	*P* value
Total	0.755 (0.422,1.089)		0.250 (0.042,0.459)	
Gender				
Male	0.768 (0.439,1.098)	–	0.255 (0.052,0.457)	–
Female	0.740 (0.390,1.092)	0.909	0.244 (0.018,0.470)	0.943
Age groups, years				
< 45	−0.019(−0.418,0.382)	–	0.006(− 0.243,0.255)	–
45–	0.701 (0.349,1.055)	0.008	0.338 (0.129,0.549)	0.046
65–	0.865 (0.463,1.268)	0.002	0.193(−0.071,0.457)	0.312
> =75	0.919 (0.567,1.273)	0.001	0.284 (0.058,0.510)	0.105
Season				
Cold	0.652 (0.193,1.114)	–	0.296(−0.010,0.603)	–
Warm	0.007(−0.711,0.731)	0.803	0.182(−0.173,0.539)	0.643
COPD	1.167 (0.820,1.515)		0.361 (0.151,0.572)	
Gender				
Male	1.192 (0.840,1.544)	–	0.390 (0.182,0.599)	–
Female	1.126 (0.747,1.507)	0.763	0.314 (0.079,0.550)	0.636
Age groups, years				
< 45	1.762 (0.769,2.765)	–	0.607(−0.038,1.257)	–
45–	1.027 (0.546,1.510)	0.193	0.326 (0.052,0.601)	0.433
65–	1.255 (0.827,1.685)	0.359	0.428 (0.174,0.683)	0.614
> =75	1.157 (0.793, 1.523)	0.263	0.342 (0.120,0.565)	0.448
Season				
Cold	1.170 (0.688,1.655)	–	0.387 (0.077, 0.699)	–
Warm	0.336 (−0.407,1.084)	0.066	0.251 (− 0.101,0.604)	0.571
Asthma	1.110 (0.513,1.710)		0.490 (0.131,0.850)	
Gender				
Male	0.915 (0.147,1.690)	–	0.356(−0.112,0.825)	–
Female	1.278 (0.527,2.034)	0.509	0.607 (0.164,1.053)	0.452
Age groups, years				
< 45	−1.176(−2.857,0.534)	–	0.098(−0.851,1.056)	–
45–	0.978 (0.165,1.799)	0.026	0.440(−0.019,0.901)	0.527
65–	1.358 (0.348,2.378)	0.012	0.360(−0.330,1.055)	0.663
> =75	2.161 (1.112,3.221)	0.001	0.935 (0.290,1.583)	0.155
Season				
Cold	1.186 (0.445,1.934)	–	0.534 (0.058,1.012)	–
Warm	−0.271(−1.500,0.974)	0.049	−0.040(− 0.670,0.594)	0.179
Pneumonia	0.842 (0.442,1.244)		0.317 (0.072,0.562)	
Gender				
Male	0.836 (0.432,1.243)	–	0.356 (0.113,0.602)	–
Female	0.850 (0.417,1.286)	0.963	0.277 (0.006,0.549)	0.672
Age groups, years				
< 45	−0.178(−0.832,0.480)		−0.036(− 0.437,0.367)	–
45–	0.966 (0.482,1.453)	0.006	0.477 (0.190,0.765)	0.042
65–	1.129 (0.613,1.648)	0.002	0.296(−0.048,0.639)	0.219
> =75	0.894 (0.482,1.308)	0.007	0.318 (0.058,0.580)	0.148
Season				
Cold	0.945 (0.422,1.470)	–	0.547 (0.201,0.895)	–
Warm	−0.312(−1.129,0.512)	0.012	−0.002(− 0.410,0.409)	0.045

*Note*: Particulate matter concentration is lag 0 for total respiratory diseases and pneumonia; lag 1 for asthma and COPD

### Exposure-response relationships

**Fig. 4:**
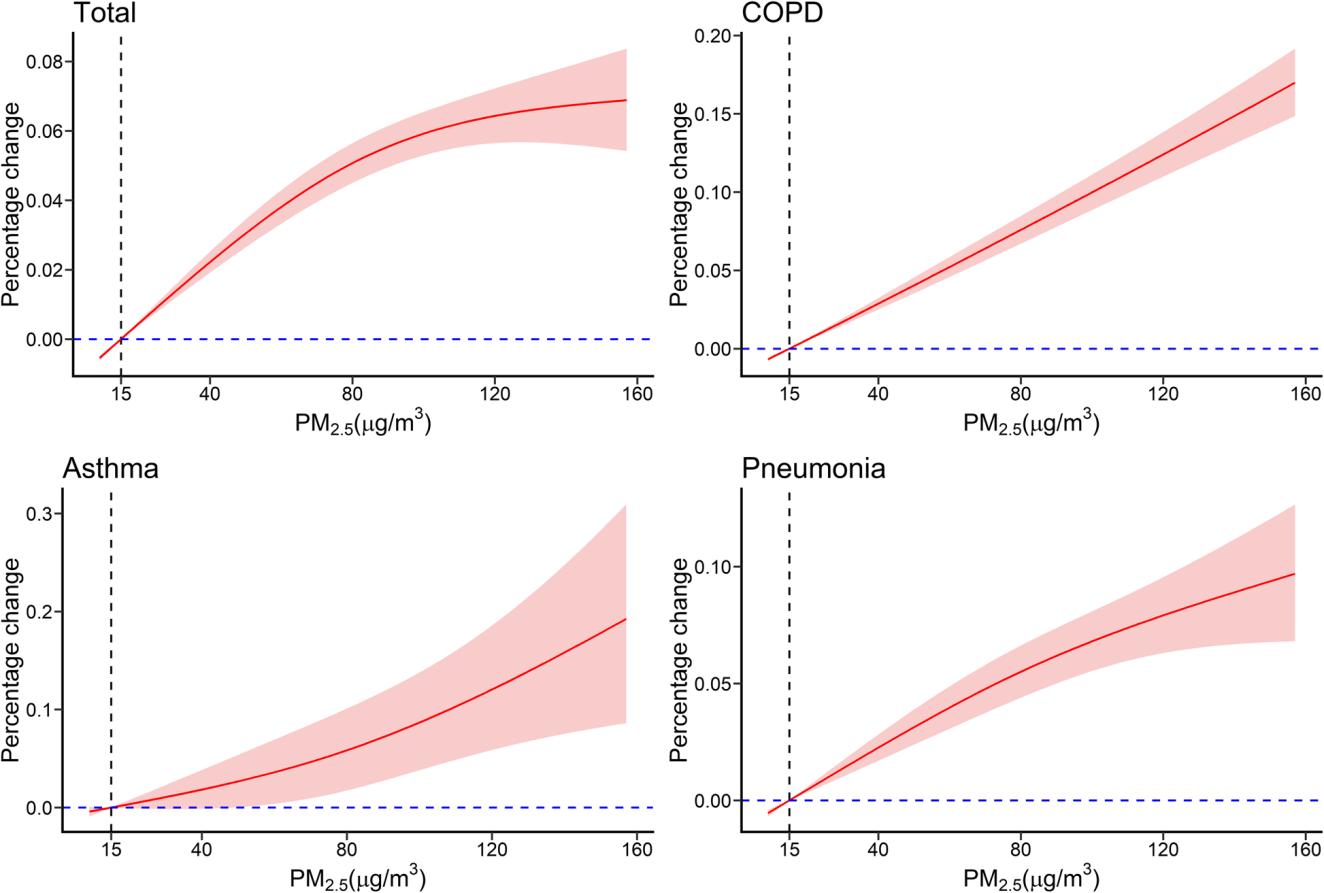
Relationship of PM_2.5_ exposure with total respiratory diseases and cause-specific respiratory diseases. The vertical line indicates the air quality standard of the WHO for daily PM_2.5_ concentration (15 μg/m^3^)

**Fig. 5:**
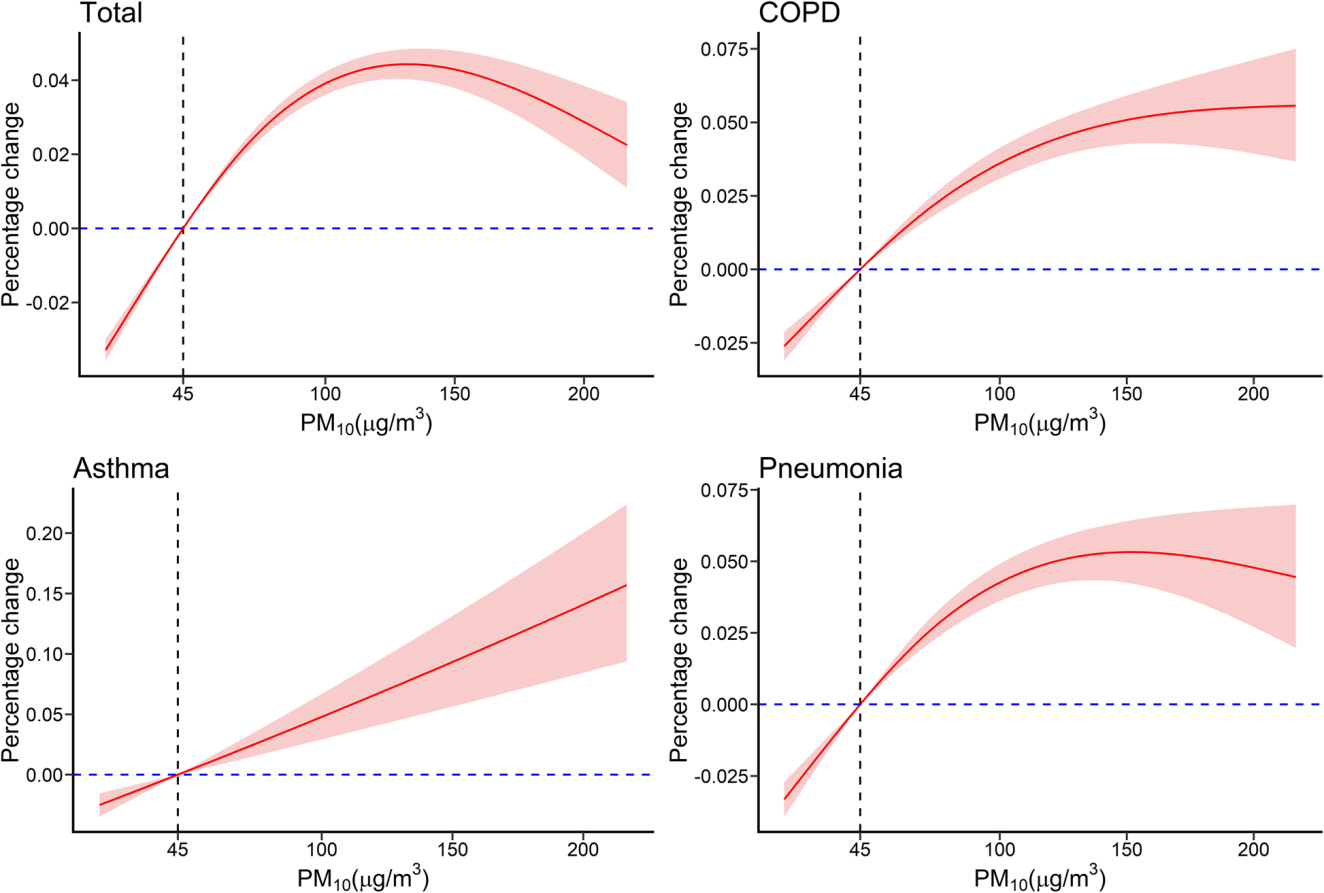
Relationship of PM_10_ exposure with total respiratory diseases and cause-specific respiratory diseases. The vertical line indicates the air quality standard of the WHO for daily PM_10_ concentration (45 μg/m^3^)

Exposure-response curves indicated that the percentage change of all types of respiratory diseases increased as PM concentrations increased (Figs. 4 and 5); these relationships were linear at low PM concentrations (0 ~ 100 μg/m^3^), but PM concentrations above 100 μg/m^3^ had less of an effect. Among the cause-specific respiratory diseases, PM_2.5_ level had a nearly linear association with COPD up to 160 μg/m^3^, PM_10_ level had a nearly linear relationship with asthma up to 200 μg/m^3^, and asthma appeared to increase exponentially with PM_2.5_ level up to 160 μg/m^3^.

### Population attributable fraction (PAF)

We also assessed the excess number of respiratory diseases due to PM_2.5_ and PM_10_ levels that exceeded the limits established by the WHO (Table S III - Table S XV). From 2013 to 2020, the overall total excess number of respiratory disease admissions was 21,678 (95% CI: 21,243, 22,112) due to PM_2.5_ and 5865 (95% CI: 5587, 6142) due to PM_10_. There were also trends of decreasing annual admissions for respiratory diseases from 2013 to 2020.

### Sensitivity analyses

We initially performed a sensitivity analysis by fitting a two-pollutant model (Table S XVI). The results indicated that the associations of PM_2.5_ and PM_10_ with total respiratory diseases and individual diseases remained statistically significant after adjusting for other pollutants, although these effect estimates were not significant after adjusting for NO_2_. Our second sensitivity analysis indicated almost no change of the effect estimates after changing the degrees of freedom for calendar time (Table S XVII). When excluding the data from 2020, the effect estimates were similar to the main analysis (Table S XVIII).

## Discussion

China is one of the most polluted countries in the world due to the rapid industrialization and urbanization [16]. In 2013, China experienced a severe air pollution event in most parts of the country, an event that was of great concern to the government. China’s State Council subsequently issued the Air Pollution Prevention and Control Plan in 2013, and strengthening of environmental protections has greatly reduced pollution in China by PM_2.5_. In response, the annual PM concentrations in Shanghai decreased markedly from 2013 to 2020. In 2019, the average PM_2.5_ concentration (35.17 μg/m^3^) was close to the national secondary standard limit of the Chinese National Ambient Air Quality Standard (35 μg/m^3^). However, the concentrations were still significantly higher than the Air Quality Guidelines 2021 for PM_2.5_ (5 μg/m^3^). The health risks from exposure to PM should not be ignored.

### Main findings and interpretation

The present ecological study found that short-term exposure to PM was significantly correlated with an increased risk of hospital admissions for total respiratory diseases and cause-specific respiratory diseases. Several previous studies also suggested an association of PM with respiratory diseases. For example, three studies [8, 17, 18] reported an association between PM and COPD and two other studies reported that short-term and long-term exposure to PM_2.5_ and PM_10_ increased the risk of COPD [19, 20]. Three other studies [21–23] examined the influence of daily PM concentrations on the number of asthma-related admissions. Li et al. [24] found a significant reduction of asthma outpatient visits during the 2008 Olympic and Paralympic Games in Beijing, which they attributed to control measures that were implemented to improve air quality. A recent national time-series study in China from 2014 to 2017 showed a short-term positive association between ambient PM concentration and hospital admissions for pneumonia in Chinese adults [25].

In line with previous studies [25, 26], we found that PM_2.5_ had a greater impact on respiratory diseases than PM_10_ at all lag structures. Compared to PM_10_, PM_2.5_ adsorbs toxic substances and heavy metals more readily due to its larger relative surface area, it remains suspended in the atmosphere for longer periods, and it enters the terminal alveoli and even the bloodstream more easily [25].

Our age-stratified analysis found a significantly greater effect of PM on total respiratory diseases, asthma, and pneumonia in patients aged 45 years or more. The effect was especially strong in those aged 65 years or above, in accordance with previous studies [25–27]. Aged people may be more vulnerable to PM_2.5_ due to their weaker immune responses [28, 29]. In addition, older people have a higher prevalence of pre-existing respiratory diseases [5].

We found a significant association of PM with respiratory diseases during the cold season, but not during the warm season, in line with previous studies of PM and respiratory diseases [30, 31]. There are several possible explanations for this finding. First, the PM_2.5_ levels due to heavy pollution were greater during the cold season than the warm season (55.42 μg/m^3^ vs. 35.41 μg/m^3^). Second, low temperatures prolong the survival of respiratory viruses in the environment [32]. Third, inhalation of cold air can inhibit the mucociliary clearance and thereby promote the spread of viruses in the respiratory tract [33].

Our analysis of exposure-response relationships indicated a linear association between PM and total respiratory diseases at lower concentrations, but a progressively weaker effect of PM on respiratory diseases at concentrations above 100 μg/m^3^. These results are similar to those of a time-series study of 245,442 respiratory visits in Nanjing, China that examined the association of PM with respiratory system diseases [10]. Tian et al. [25] also reported a nonlinear relationship between PM_2.5_ level and pneumonia, with a weaker effect at higher concentrations. This nonlinear relationship may be because people avoid spending time outside or wear a dust mask when outside when the air is heavily polluted. The exposure-response curves suggested no discernible thresholds of PM. Exposure to PM_2.5_ and PM_10_ even at concentrations below the current regulatory limits is associated with increases in daily admissions, suggesting that tightening the current air quality guidelines of PM for greater public health benefit is necessary.

Our two-pollutant model indicated the associations between PM and respiratory diseases remained positive, but not significant after adjusting for NO_2_. Previous studies reported similar results. For example, Tian et al. [25] reported no significant association between PM and pneumonia after controlling for NO_2_. Liu et al. [34] suggested that the effect of PM on respiratory admissions decreased dramatically and was no longer significant after controlling for NO_2_. Tsai et al. [35] found that the adverse effect of PM_2.5_ was not statistically significant after adjusting for NO_2_ or CO. It is possible that a high correlation among pollutants weakened the effect estimation. Therefore, the independent effect of PM on respiratory diseases should be examined.

### Potential mechanism

The potential biological mechanism linking the exposure to PM and hospital admissions for respiratory diseases are not fully understood, but there are several plausible mechanisms. First, exposure to PM may lead to oxidative stress. For example, PM_2.5_ can easily adsorb organic pollutants, such as polycyclic aromatic hydrocarbons and heavy metals, which promote the production of reactive oxygen species in lung cells, consume antioxidant substances, trigger oxidative stress reactions, and induce lung inflammation [36]. A meta-analysis [37] of epidemiology studies suggested that short-term exposure to PM_2.5_ was associated with an increased level of malondialdehyde, a biomarker of oxidative stress. Second, PM_2.5_ induction of a local or systematic inflammatory reaction is the major pathologic basis for the onset and progression of several related diseases [38]. Evidence from a meta-analysis [39] also showed that PM exposure increased the levels of fibrinogen and TNF-α.

### Limitations

Compared with previous studies, we used a larger sample size and examined a longer time period, resulting in greater statistical power. However, this study had several limitations. First, our study was a one-city observational study, and multi-city studies are necessary to validate our conclusions. Second, due to unavailability of residential location where each patient lived, we used average concentrations of daily air pollutants from fixed environmental monitoring stations as an indicator of exposure. This might result in measurement error, which tends to be non-differential and underestimate the effect of the PM effects [40]. Previous studies used satellite-derived PM_2.5_ concentrations based on modeling techniques to represent individual exposure levels and to examine the potential effect on several diseases, including asthma [41] and poor birth outcome [42]. Third, potential confounders, including education level, occupation, socioeconomic status (e.g., family income), and individual behaviors (e.g., smoking status), were not available from the medical insurance system, making stratified analysis impossible. Finally, this study was a time-series ecological investigation of the associations of PM with health outcomes, and cannot make definitive conclusions regarding causality. Our results also could have been affected by the ecological fallacy.

## Conclusion

The main result of this study is that short-term exposure to PM in Shanghai was significantly associated with hospital admissions for total respiratory diseases and cause-specific respiratory diseases — COPD, asthma, and pneumonia. These associations were especially stronger for patients more than 45 years old and during the cold season. PM level had a nearly linear relationship with total respiratory diseases at low concentrations (0 ~ 100 μg/m^3^), but had a weaker as PM concentrations increased. Reducing atmospheric PM concentrations may reduce hospital admissions for respiratory diseases. These findings have important implications for policymakers to take concrete actions to reduce atmospheric PM concentrations. Further studies conducted on nationwide regions are required to validate our results.

## Supplementary Information

**Additional file 1: Table S I.** The annual average concentrations of PMs in Shanghai. **Table S II.** Spearman correlation coefficients among air pollution variables and meteorological factors. **Table S III.** The attributable number of respiratory diseases admissions due to exceeding PM_2.5_ concentrations. **Table S IV.** The attributable number of respiratory diseases admissions due to exceeding PM_10_ concentrations. **Table S V.** The attributable number of respiratory diseases admissions due to exceeding PM_2.5_ concentrations in males. **Table S VI.** The attributable number of respiratory diseases admissions due to exceeding PM_2.5_ concentrations in females. **Table S VII.** The attributable number of respiratory diseases admissions due to exceeding PM_10_ concentrations in males. **Table S VIII.** The attributable number of respiratory diseases admissions due to exceeding PM_10_ concentrations in females. **Table S III.** The attributable number of respiratory diseases admissions due to exceeding PM_2.5_ concentrations in < 45 years. **Table S IX.** The attributable number of respiratory diseases admissions due to exceeding PM_2.5_ concentrations in 45 ~ 64 years. **Table S X.** The attributable number of respiratory diseases admissions due to exceeding PM_2.5_ concentrations in 65 ~ 74 years. **Table S XI.** The attributable number of respiratory diseases admissions due to exceeding PM_2.5_ concentrations in ≥75 years. **Table S XII.** The attributable number of respiratory diseases admissions due to exceeding PM_10_ concentrations in < 45 years. **Table S XIII.** The attributable number of respiratory diseases admissions due to exceeding PM_10_ concentrations in 45 ~ 64 years. **Table S XIV.** The attributable number of respiratory diseases admissions due to exceeding PM_10_ concentrations in 65 ~ 74 years. **Table S XV.** The attributable number of respiratory diseases admissions due to exceeding PM_10_ concentrations in ≥75 years. **Table S XVI.** Percentage change with 95% confidence interval in hospital admissions for respiratory diseases per 10 μg/m^3^ increase in concentrations of PM_2.5_ and PM_10_ using two-pollutant models. **Table S XVII.** Percentage change with 95% confidence interval in hospital admissions for respiratory diseases associated with a 10 μg/m^3^ increase in concentrations of PM_2.5_ and PM_10_ through changing degrees of freedom for the calendar time. **Table S XVIII.** Percentage change with 95% confidence interval in hospital admissions for respiratory diseases associated with a 10 μg/m3 increase in concentrations of PM_2.5_ and PM_10_ through excluding the data from 2020 due to the coronavirus disease 2019 pandemic. **Fig. S I.** Percentage change with 95% confidence interval in hospital admissions for respiratory diseases per 10 μg/m^3^ increase in concentrations of PM_2.5_ stratified by genders. All the models were adjusted by public holidays, DOW, and calendar day. **Fig. S II.** Percentage change with 95% confidence interval in hospital admissions for respiratory diseases per 10 μg/m^3^ increase in concentrations of PM_10_ stratified by genders. All the models were adjusted with public holidays, DOW, and calendar day. **Fig. S III.** Percentage change with 95% confidence interval in hospital admissions for respiratory diseases per 10 μg/m^3^ increase in concentrations of PM_2.5_ stratified by age groups. All the models were adjusted with public holidays, DOW, and calendar day. **Fig. S IV.** Percentage change with 95% confidence interval in hospital admissions for respiratory diseases per 10 μg/m^3^ increase in concentrations of PM_10_ stratified by age groups. All the models were adjusted with public holidays, DOW, and calendar day. **Fig. S V.** Percentage change with 95% confidence interval in hospital admissions for respiratory diseases per 10 μg/m^3^ increase in concentrations of PM_2.5_ stratified by season. All the models were adjusted with public holidays, DOW, and calendar day. **Fig. S VI**. Percentage change with 95% confidence interval in hospital admissions for respiratory diseases per 10 μg/m^3^ increase in concentrations of PM_10_ stratified by season. All the models were adjusted with public holidays, DOW, and calendar day.

## Data Availability

The data that support the findings of this study are available from the corresponding author upon reasonable request.
