# Supplementary material for: Effects of particulate matter on hospital admissions for respiratory diseases: an ecological study based on 12.5 years of time series data in Shanghai
Source: Environ Health. 2022 Jan 13;21:12. doi: 10.1186/s12940-021-00828-6 (PMC8756174; doi:10.1186/s12940-021-00828-6)
Supplement: Supplementary file 1 — Additional file 1: Table S I. The annual average concentrations of PMs in Shanghai. Table S II. Spearman correlation coefficients among air pollution variables and meteorological factors. Table S III. The attributable number of respiratory diseases admissions due to exceeding PM2.5 concentrations. Table S IV. The attributable number of respiratory diseases admissions due to exceeding PM10 concentrations. Table S V. The attributable number of respiratory diseases admissions due to exceeding PM2.5 concentrations in males. Table S VI. The attributable number of respiratory diseases admissions due to exceeding PM2.5 concentrations in females. Table S VII. The attributable number of respiratory diseases admissions due to exceeding PM10 concentrations in males. Table S VIII. The attributable number of respiratory diseases admissions due to exceeding PM10 concentrations in females. Table S III. The attributable number of respiratory diseases admissions due to exceeding PM2.5 concentrations in < 45 years. Table S IX. The attributable number of respiratory diseases admissions due to exceeding PM2.5 concentrations in 45 ~ 64 years. Table S X. The attributable number of respiratory diseases admissions due to exceeding PM2.5 concentrations in 65 ~ 74 years. Table S XI. The attributable number of respiratory diseases admissions due to exceeding PM2.5 concentrations in ≥75 years. Table S XII. The attributable number of respiratory diseases admissions due to exceeding PM10 concentrations in < 45 years. Table S XIII. The attributable number of respiratory diseases admissions due to exceeding PM10 concentrations in 45 ~ 64 years. Table S XIV. The attributable number of respiratory diseases admissions due to exceeding PM10 concentrations in 65 ~ 74 years. Table S XV. The attributable number of respiratory diseases admissions due to exceeding PM10 concentrations in ≥75 years. Table S XVI. Percentage change with 95% confidence interval in hospital admissions for respiratory diseases pe [file 12940_2021_828_MOESM1_ESM.docx]

**Effects of** **particulate matter on hospital admissions for respiratory diseases: an ecological study based on 12.5 years of time series data in Shanghai**

Wenjia Peng ^a^, Hao Li ^a^, Li Peng ^b^, Ying Wang ^a,c*^, Weibing Wang ^a,b,d**^

Table S I The annual average concentrations of PMs in Shanghai

| Calendar year | PM_2.5_ | PM_10_ |
| --- | --- | --- |
| 2008 | - | 82.41±44.35 |
| 2009 | - | 79.00±46.31 |
| 2010 | - | 78.41±60.24 |
| 2011 | - | 75.21±58.55 |
| 2012 | - | 71.33±40.90 |
| 2013 | 60.70±43.80 | 80.85±52.60 |
| 2014 | 51.89±32.08 | 71.21±41.27 |
| 2015 | 50.36±33.61 | 65.19±38.04 |
| 2016 | 42.40±28.23 | 56.11±31.68 |
| 2017 | 36.56±22.21 | 53.18±27.03 |
| 2018 | 34.18±25.04 | 48.21±27.30 |
| 2019 | 35.17±21.74 | 46.90±27.89 |
| 2020 (Jan 1^st^ – July 31^st^) | 34.73±21.13 | 41.09±19.54 |

Table S II Spearman correlation coefficients among air pollution variables and meteorological factors

| Air pollution | PM_10_ | PM_2.5_ | NO_2_ | SO_2_ | O_3_ | CO | Temp | RH |
| --- | --- | --- | --- | --- | --- | --- | --- | --- |
| PM_10_ | 1.000 | - | - | - | - | - | - | - |
| PM_2.5_ | 0.872** | 1.000 | - | - | - | - | - | - |
| NO_2_ | 0.685** | 0.721** | 1.000 | - | - | - | - | - |
| SO_2_ | 0.655** | 0.729** | 0.683** | 1.000 | - | - | - | - |
| O_3_ | 0.086** | 0.027 | -0.181** | -0.112** | 1.000 | - | - | - |
| CO | 0.759** | 0.879** | 0.760** | 0.691** | -0.130** | 1.000 | - | - |
| Temp | -0.229** | -0.311** | -0.412** | -0.334** | 0.533** | -0.329** | 1.000 | - |
| RH | -0.365** | -0.142** | -0.182** | -0.356** | -0.328** | 0.035 | 0.154** | 1.000 |

Note: Temp, Temperature; RH, Relative humidity

Table S III The attributable number of respiratory diseases admissions due to exceeding PM_2.5_ concentrations

| Calendar year | Respiratory diseases | COPD | Asthma | Pneumonia |
| --- | --- | --- | --- | --- |
| 2013 | 5035(4990,5081) | 2670(2655,2686) | 120(118,121) | 1210(1197,1222) |
| 2014 | 4333(4284,4382) | 2239(2223,2255) | 109(108,111) | 1033(1021,1046) |
| 2015 | 4681(4627,4735) | 2203(2186,2220) | 117(115,119) | 1205(1190,1220) |
| 2016 | 4529(4464,4595) | 2107(2088,2127) | 101(99,103) | 1146(1129,1164) |
| 2017 | 3526(3458,3595) | 1591(1571,1612) | 83(81,85) | 906(887,925) |
| 2018 | 3035(2973,3097) | 1468(1448,1488) | 73(71,75) | 941(920,962) |
| 2019 | 3345(3279,3411) | 1528(1508,1547) | 81(79,83) | 1092(1069,1116) |
| 2020 (Jan 1 – July 31) | 1030(1010,1050) | 477(471,483) | 27(26,28) | 327(321,334) |

Table S IV The attributable number of respiratory diseases admissions due to exceeding PM_10_ concentrations

| Calendar year | Respiratory diseases | COPD | Asthma | Pneumonia |
| --- | --- | --- | --- | --- |
| 2008 | 1065(1043,1087) | 669(659,678) | 39(38,39) | 268(263,273) |
| 2009 | 1153(1128,1179) | 745(734,757) | 41(40,42) | 293(287,300) |
| 2010 | 1211(1184,1238) | 767(755,778) | 41(40,42) | 314(308,321) |
| 2011 | 1024(998,1049) | 619(609,629) | 35(34,36) | 264(257,270) |
| 2012 | 988(960,1017) | 546(535,557) | 33(32,34) | 258(251,265) |
| 2013 | 1432(1403,1461) | 739(729,748) | 47(46,48) | 391(383,399) |
| 2014 | 1156(1124,1188) | 571(560,581) | 39(38,40) | 309(301,317) |
| 2015 | 1119(1084,1154) | 503(493,514) | 38(37,39) | 327(317,336) |
| 2016 | 992(950,1034) | 443(430,455) | 29(28,30) | 286(275,297) |
| 2017 | 749(705,793) | 320(307,333) | 24(23,25) | 221(209,233) |
| 2018 | 614(574,654) | 281(268,293) | 20(19,21) | 218(205,231) |
| 2019 | 572(530,614) | 250(238,262) | 19(17,20) | 213(198,227) |
| 2020 (Jan 1 – July 31) | 113(101,126) | 49(46,53) | 4(4,5) | 39(35,43) |

Table S V The attributable number of respiratory diseases admissions due to exceeding PM_2.5_ concentrations in male

| Calendar year | Respiratory diseases | COPD | Asthma | Pneumonia |
| --- | --- | --- | --- | --- |
| 2013 | 2893(2868,2919) | 1685(1675,1694) | 46(46,47) | 607(600,613) |
| 2014 | 2442(2415,2470) | 1402(1391,1412) | 43(42,44) | 501(495,507) |
| 2015 | 2627(2598,2657) | 1389(1379,1400) | 45(44,46) | 587(580,595) |
| 2016 | 2489(2454,2524) | 1333(1321,1345) | 40(39,42) | 543(534,551) |
| 2017 | 1922(1885,1958) | 997(984,1009) | 32(30,33) | 421(412,430) |
| 2018 | 1681(1647,1715) | 931(918,943) | 26(25,27) | 432(423,442) |
| 2019 | 1865(1829,1901) | 987(974,1000) | 29(28,30) | 506(495,518) |
| 2020 (Jan 1 – July 31) | 593(582,604) | 308(304,312) | 10(10,11) | 162(158,165) |

Table S VI The attributable number of respiratory diseases admissions due to exceeding PM_2.5_ concentrations in female

| Calendar year | Respiratory diseases | COPD | Asthma | Pneumonia |
| --- | --- | --- | --- | --- |
| 2013 | 2150(2129,2171) | 985(979,991) | 73(72,74) | 604(598,611) |
| 2014 | 1896(1873,1919) | 836(830,843) | 66(65,67) | 534(527,541) |
| 2015 | 2059(2033,2085) | 813(806,820) | 72(71,73) | 619(611,627) |
| 2016 | 2044(2012,2075) | 774(766,782) | 60(59,61) | 605(595,615) |
| 2017 | 1607(1573,1640) | 594(585,602) | 51(50,52) | 487(476,497) |
| 2018 | 1356(1327,1386) | 537(529,545) | 48(47,49) | 510(498,522) |
| 2019 | 1484(1452,1515) | 542(534,550) | 53(51,54) | 588(574,601) |
| 2020 (Jan 1 – July 31) | 439(430,448) | 169(166,171) | 17(16,17) | 166(163,170) |

Table S VII The attributable number of respiratory diseases admissions due to exceeding PM_10_ concentrations in male

| Calendar year | Respiratory diseases | COPD | Asthma | Pneumonia |
| --- | --- | --- | --- | --- |
| 2008 | 634(621,646) | 456(450,461) | 14(13,14) | 158(156,161) |
| 2009 | 671(657,685) | 495(488,502) | 14(14,15) | 167(164,170) |
| 2010 | 700(685,715) | 501(494,508) | 14(14,15) | 180(176,183) |
| 2011 | 597(583,611) | 413(407,420) | 12(12,13) | 152(149,155) |
| 2012 | 569(553,584) | 362(355,368) | 12(11,12) | 147(144,150) |
| 2013 | 823(807,839) | 491(485,497) | 15(15,16) | 221(217,225) |
| 2014 | 652(635,669) | 378(372,385) | 13(13,14) | 169(165,173) |
| 2015 | 627(609,646) | 335(329,342) | 12(12,13) | 180(175,185) |
| 2016 | 545(523,567) | 296(288,303) | 10(10,11) | 153(147,158) |
| 2017 | 410(387,433) | 213(205,220) | 8(7,9) | 116(111,122) |
| 2018 | 341(320,362) | 188(181,196) | 6(5,7) | 114(108,120) |
| 2019 | 317(295,340) | 170(162,177) | 6(5,6) | 111(104,118) |
| 2020 (Jan 1 – July 31) | 66(59,73) | 34(32,36) | 1(1,2) | 22(20,24) |

Table S VIII The attributable number of respiratory diseases admissions due to exceeding PM_10_ concentrations in female

| Calendar year | Respiratory diseases | COPD | Asthma | Pneumonia |
| --- | --- | --- | --- | --- |
| 2008 | 431(421,441) | 215(211,219) | 25(24,25) | 111(109,114) |
| 2009 | 481(469,493) | 249(244,254) | 27(26,27) | 127(124,131) |
| 2010 | 509(497,522) | 263(258,268) | 27(26,27) | 136(132,139) |
| 2011 | 426(414,437) | 205(201,210) | 22(22,23) | 112(109,116) |
| 2012 | 418(405,432) | 184(179,188) | 21(21,22) | 112(108,116) |
| 2013 | 607(594,621) | 247(243,251) | 32(31,32) | 170(166,174) |
| 2014 | 502(487,517) | 192(187,196) | 26(25,26) | 139(135,144) |
| 2015 | 490(473,507) | 168(163,172) | 25(25,26) | 146(141,152) |
| 2016 | 445(424,466) | 147(142,152) | 19(18,19) | 132(125,138) |
| 2017 | 338(316,360) | 107(102,113) | 16(15,17) | 103(96,110) |
| 2018 | 272(253,291) | 93(87,98) | 14(13,15) | 102(95,110) |
| 2019 | 254(233,274) | 81(76,86) | 13(12,14) | 100(92,109) |
| 2020 (Jan 1 – July 31) | 47(42,53) | 16(14,17) | 3(3,3) | 17(15,19) |

Table S III The attributable number of respiratory diseases admissions due to exceeding PM_2.5_ concentrations in <45 years

| Calendar year | Respiratory diseases | COPD | Asthma | Pneumonia |
| --- | --- | --- | --- | --- |
| 2013 | 0(0,0) | 100(99,101) | 0(0,0) | 0(0,0) |
| 2014 | 0(0,0) | 81(80,82) | 0(0,0) | 0(0,0) |
| 2015 | 0(0,0) | 56(55,57) | 0(0,0) | 0(0,0) |
| 2016 | 0(0,0) | 38(38,39) | 0(0,0) | 0(0,0) |
| 2017 | 0(0,0) | 31(30,32) | 0(0,0) | 0(0,0) |
| 2018 | 0(0,0) | 30(29,31) | 0(0,0) | 0(0,0) |
| 2019 | 0(0,0) | 39(38,40) | 0(0,0) | 0(0,0) |
| 2020 (Jan 1 – July 31) | 0(0,0) | 12(12,12) | 0(0,0) | 0(0,0) |

Table S IX The attributable number of respiratory diseases admissions due to exceeding PM_2.5_ concentrations in 45~64 years

| Calendar year | Respiratory diseases | COPD | Asthma | Pneumonia |
| --- | --- | --- | --- | --- |
| 2013 | 1129(1118,1141) | 360(356,363) | 55(54,56) | 329(326,333) |
| 2014 | 966(954,979) | 307(303,310) | 46(45,47) | 289(285,293) |
| 2015 | 1015(1001,1028) | 284(281,288) | 50(49,51) | 332(327,336) |
| 2016 | 674(663,685) | 148(146,151) | 27(26,28) | 208(204,211) |
| 2017 | 546(534,559) | 123(120,126) | 22(22,23) | 171(168,175) |
| 2018 | 483(471,494) | 125(122,128) | 21(21,22) | 199(194,204) |
| 2019 | 557(544,570) | 143(140,146) | 23(22,24) | 241(235,246) |
| 2020 (Jan 1 – July 31) | 172(168,176) | 51(50,52) | 8(8,8) | 69(67,70) |

Table S X The attributable number of respiratory diseases admissions due to exceeding PM_2.5_ concentrations in 65~74 years

| Calendar year | Respiratory diseases | COPD | Asthma | Pneumonia |
| --- | --- | --- | --- | --- |
| 2013 | 866(858,874) | 476(473,480) | 25(24,25) | 242(239,244) |
| 2014 | 774(764,783) | 417(414,421) | 27(26,27) | 217(215,220) |
| 2015 | 891(880,901) | 416(412,420) | 29(29,30) | 281(278,285) |
| 2016 | 1098(1081,1115) | 442(437,447) | 44(43,45) | 342(337,347) |
| 2017 | 879(861,898) | 346(341,351) | 35(34,36) | 280(275,286) |
| 2018 | 798(781,815) | 360(354,365) | 33(32,34) | 305(298,311) |
| 2019 | 924(905,943) | 411(405,417) | 35(34,36) | 358(351,365) |
| 2020 (Jan 1 – July 31) | 291(285,297) | 139(137,141) | 13(12,13) | 104(102,106) |

Table S XI The attributable number of respiratory diseases admissions due to exceeding PM_2.5_ concentrations in ≥75 years

| Calendar year | Respiratory diseases | COPD | Asthma | Pneumonia |
| --- | --- | --- | --- | --- |
| 2013 | 2976(2953,2999) | 1737(1727,1748) | 45(44,45) | 636(630,642) |
| 2014 | 2572(2547,2597) | 1436(1425,1448) | 41(41,42) | 538(532,544) |
| 2015 | 2771(2743,2798) | 1443(1432,1455) | 47(46,47) | 622(615,630) |
| 2016 | 2916(2880,2952) | 1489(1475,1504) | 50(49,51) | 644(635,654) |
| 2017 | 2218(2181,2254) | 1100(1085,1114) | 42(41,43) | 493(484,503) |
| 2018 | 1905(1872,1938) | 963(950,977) | 30(30,31) | 478(468,488) |
| 2019 | 1941(1909,1974) | 949(936,962) | 34(33,35) | 513(502,523) |
| 2020 (Jan 1 – July 31) | 596(586,606) | 279(275,283) | 10(10,10) | 162(158,165) |

Table S XII The attributable number of respiratory diseases admissions due to exceeding PM_10_ concentrations in <45 years

| Calendar year | Respiratory diseases | COPD | Asthma | Pneumonia |
| --- | --- | --- | --- | --- |
| 2008 | 2(0,5) | 18(18,19) | 1(1,1) | 0(0,0) |
| 2009 | 2(0,5) | 28(28,29) | 1(1,1) | 0(0,0) |
| 2010 | 2(0,5) | 30(29,31) | 1(1,1) | 0(0,0) |
| 2011 | 2(-1,5) | 31(30,32) | 1(1,1) | 0(0,0) |
| 2012 | 3(-1,6) | 28(27,29) | 1(1,1) | 0(0,0) |
| 2013 | 4(-1,8) | 36(35,37) | 1(1,1) | 0(0,0) |
| 2014 | 3(-2,8) | 23(23,24) | 1(1,1) | 0(0,0) |
| 2015 | 3(-2,8) | 13(13,14) | 1(0,1) | 0(0,0) |
| 2016 | 2(-3,7) | 9(9,10) | 0(0,1) | 0(0,0) |
| 2017 | 2(-4,7) | 7(7,8) | 0(0,1) | 0(0,0) |
| 2018 | 1(-3,5) | 6(5,7) | 0(0,1) | 0(0,0) |
| 2019 | 1(-4,7) | 8(7,8) | 0(0,1) | 0(0,0) |
| 2020 (Jan 1 – July 31) | 0(-1,2) | 1(1,2) | 0(0,0) | 0(0,0) |

Table S XIII The attributable number of respiratory diseases admissions due to exceeding PM_10_ concentrations in 45~64 years

| Calendar year | Respiratory diseases | COPD | Asthma | Pneumonia |
| --- | --- | --- | --- | --- |
| 2008 | 334(328,339) | 86(84,87) | 18(17,18) | 100(99,102) |
| 2009 | 371(365,377) | 103(101,105) | 20(19,20) | 119(117,121) |
| 2010 | 381(374,387) | 103(101,105) | 20(19,20) | 119(117,121) |
| 2011 | 316(310,322) | 78(76,80) | 16(15,16) | 101(99,103) |
| 2012 | 309(303,316) | 71(69,73) | 15(15,16) | 90(88,91) |
| 2013 | 464(456,471) | 104(102,106) | 22(21,22) | 139(137,142) |
| 2014 | 373(365,381) | 80(78,82) | 17(16,17) | 114(112,116) |
| 2015 | 349(341,357) | 66(63,68) | 17(16,17) | 118(116,121) |
| 2016 | 210(203,217) | 31(30,32) | 8(8,8) | 67(65,69) |
| 2017 | 168(160,175) | 25(23,26) | 7(6,7) | 55(53,57) |
| 2018 | 140(133,147) | 25(23,26) | 6(5,6) | 60(57,63) |
| 2019 | 136(129,144) | 24(22,26) | 6(5,6) | 60(57,63) |
| 2020 (Jan 1 – July 31) | 28(26,31) | 6(5,6) | 1(1,1) | 10(10,11) |

Table S XIV The attributable number of respiratory diseases admissions due to exceeding PM_10_ concentrations in 65~74 years

| Calendar year | Respiratory diseases | COPD | Asthma | Pneumonia |
| --- | --- | --- | --- | --- |
| 2008 | 146(141,151) | 158(155,160) | 5(4,5) | 44(42,45) |
| 2009 | 147(142,153) | 162(159,164) | 5(4,5) | 44(43,46) |
| 2010 | 144(138,149) | 154(152,156) | 4(4,4) | 44(43,46) |
| 2011 | 122(117,127) | 122(120,124) | 4(4,4) | 40(38,41) |
| 2012 | 113(108,118) | 109(107,112) | 3(3,4) | 38(36,39) |
| 2013 | 167(162,173) | 144(142,146) | 6(5,6) | 56(54,57) |
| 2014 | 139(133,145) | 116(114,118) | 6(6,6) | 46(44,48) |
| 2015 | 144(137,151) | 105(103,107) | 6(5,6) | 54(52,56) |
| 2016 | 162(151,174) | 102(99,105) | 8(7,8) | 60(57,64) |
| 2017 | 127(114,139) | 78(75,81) | 6(6,7) | 48(44,52) |
| 2018 | 110(98,122) | 76(73,80) | 5(5,6) | 49(45,54) |
| 2019 | 106(94,119) | 73(70,77) | 5(4,6) | 49(44,53) |
| 2020 (Jan 1 – July 31) | 22(18,26) | 16(15,17) | 1(1,2) | 9(7,10) |

Table S XV The attributable number of respiratory diseases admissions due to exceeding PM_10_ concentrations in ≥75 years

| Calendar year | Respiratory diseases | COPD | Asthma | Pneumonia |
| --- | --- | --- | --- | --- |
| 2008 | 608(596,620) | 407(401,413) | 15(15,16) | 128(125,130) |
| 2009 | 667(653,681) | 452(445,460) | 14(14,14) | 137(134,140) |
| 2010 | 728(712,743) | 478(470,486) | 17(17,18) | 158(155,162) |
| 2011 | 611(597,626) | 389(381,396) | 16(15,16) | 132(129,135) |
| 2012 | 570(554,585) | 339(332,347) | 14(14,15) | 131(128,135) |
| 2013 | 795(780,810) | 455(448,462) | 18(17,18) | 195(191,199) |
| 2014 | 644(628,661) | 351(344,358) | 15(14,15) | 153(149,157) |
| 2015 | 627(609,645) | 316(309,324) | 15(15,15) | 161(156,166) |
| 2016 | 604(581,628) | 300(291,309) | 15(14,15) | 154(148,161) |
| 2017 | 441(417,465) | 211(201,220) | 12(12,13) | 114(108,120) |
| 2018 | 362(341,384) | 176(167,184) | 8(8,8) | 107(100,113) |
| 2019 | 313(292,335) | 149(141,157) | 7(7,8) | 96(89,103) |
| 2020 (Jan 1 – July 31) | 61(54,67) | 27(25,29) | 2(2,2) | 19(17,21) |

Table S XVI Percentage change with 95% confidence interval in hospital admissions for respiratory diseases per 10 μg/m^3^ increase in concentrations of PM_2.5_ and PM_10_ using two-pollutant models.

| PM | Two-pollutant | Respiratory | COPD | Asthma | Pneumonia |
| --- | --- | --- | --- | --- | --- |
| PM_2.5_ | - | 0.755(0.422,1.089) | 1.167 (0.820,1.515) | 1.110(0.513,1.710) | 0.842(0.442,1.244) |
|  | +NO_2_ | 0.231(-0.227,0.691) | 0.472(-0.002,0.948) | 0.430(-0.205,1.070) | 0.489(-0.061,1.042) |
|  | +SO_2_ | 1.310(0.856,1.765) | 1.378(0.904,1.853) | 0.798(0.163,1.437) | 1.912(1.372,2.455) |
|  | +O_3_ | 0.673(0.333,1.016) | 1.052(0.702,1.403) | 1.172(0.571,1.777) | 0.768(0.357,1.181) |
|  | +CO | 1.493(0.824,2.166) | 2.092(1.388,2.801) | 0.864(0.209,1.524) | 2.069(1.263,2.882) |
| PM_10_ | - | 0.250(0.042,0.459) | 0.361(0.151,0.572) | 0.490(0.131,0.850) | 0.317(0.072,0.562) |
|  | +NO_2_ | 0.102(-0.172,0.376) | 0.154(-0.124,0.434) | 0.171(-0.214,0.558) | 0.209(-0.112,0.530) |
|  | +SO_2_ | 0.585(0.320,0.851) | 0.749(0.481,1.017) | 0.366(-0.013,0.746) | 1.002(0.698,1.306) |
|  | +O_3_ | 0.526(0.227,0.826) | 0.893(0.587,1.201) | 1.251(0.732,1.772) | 0.593(0.231,0.956) |
|  | +CO | 0.690(0.210,1.173) | 1.156(0.652,1.663) | 1.072(0.514,1.633) | 0.964(0.388,1.543) |

Note: Particulate matter concentration is lag 0 for all respiratory diseases and pneumonia; lag 1 for asthma and COPD.

Table S XVII Percentage change with 95% confidence interval in hospital admissions for respiratory diseases associated with a 10μg/m^3^ increase in concentrations of PM_2.5_ and PM_10_ through changing degrees of freedom for the calendar time.

| Respiratory diseases | PM_2.5_ | PM_10_ |
| --- | --- | --- |
| All |  |  |
| 5 | 0.755 (0.422,1.089) | 0.250 (0.042,0.459) |
| 6 | 0.755 (0.422,1.089) | 0.250 (0.042,0.459) |
| **7** | 0.755 (0.422,1.089) | 0.250 (0.042,0.459) |
| 8 | 0.755 (0.422,1.089) | 0.250 (0.042,0.459) |
| 9 | 0.755 (0.422,1.089) | 0.250 (0.042,0.459) |
| COPD |  |  |
| 5 | 1.139 (0.795,1.483) | 0.338 (0.126,0.551) |
| 6 | 1.139 (0.795,1.483) | 0.338 (0.126,0.551) |
| **7** | 1.139 (0.795,1.483) | 0.338 (0.126,0.551) |
| 8 | 1.139 (0.795,1.483) | 0.338 (0.126,0.551) |
| 9 | 1.139 (0.795,1.483) | 0.338 (0.126,0.551) |
| Asthma |  |  |
| 5 | 1.110 (0.513,1.710) | 0.490 (0.131,0.850) |
| 6 | 1.110 (0.513,1.710) | 0.490 (0.131,0.850) |
| **7** | 1.110 (0.513,1.710) | 0.490 (0.131,0.850) |
| 8 | 1.110 (0.513,1.710) | 0.490 (0.131,0.850) |
| 9 | 1.110 (0.513,1.710) | 0.490 (0.131,0.850) |
| Pneumonia |  |  |
| 5 | 0.842 (0.442,1.244) | 0.317 (0.072,0.562) |
| 6 | 0.842 (0.442,1.244) | 0.317 (0.072,0.562) |
| **7** | 0.842 (0.442,1.244) | 0.317 (0.072,0.562) |
| 8 | 0.842 (0.442,1.244) | 0.317 (0.072,0.562) |
| 9 | 0.842 (0.442,1.244) | 0.317 (0.072,0.562) |

Note: Particulate matter concentration is lag 0 for all respiratory diseases and pneumonia; lag 1 for asthma and COPD.

Table S XVIII Percentage change with 95% confidence interval in hospital admissions for respiratory diseases associated with a 10μg/m3 increase in concentrations of PM2.5 and PM10 through excluding the data from 2020 due to the coronavirus disease 2019 pandemic

| Respiratory diseases | PM_2.5_ | PM_10_ |
| --- | --- | --- |
| All | 0.785 (0.486, 1.086) | 0.234 (0.046, 0.423) |
| COPD | 1.197 (0.879, 1.516) | 0.336 (0.137, 0.535) |
| Asthma | 1.197 (0.879, 1.516) | 0.467 (0.114, 0.821) |
| Pneumonia | 0.885 (0.538, 1.233) | 0.322 (0.106, 0.539) |

Note: Particulate matter concentration is lag 0 for all respiratory diseases and pneumonia; lag 1 for asthma and COPD.


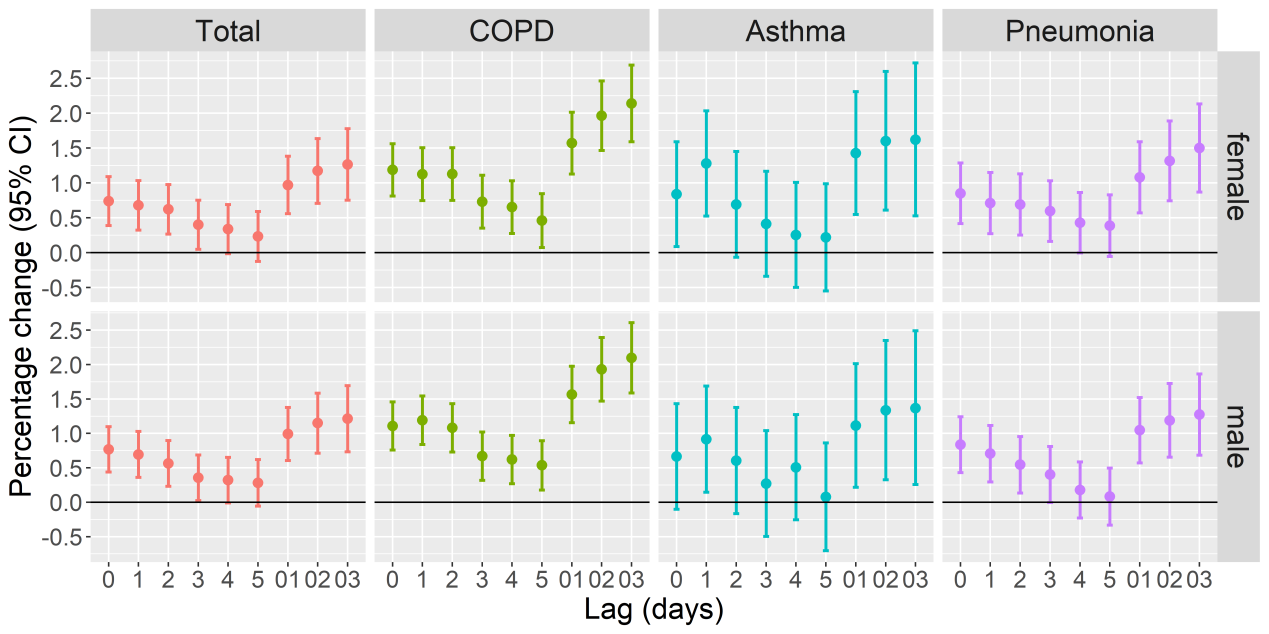


Fig. S I Percentage change with 95% confidence interval in hospital admissions for respiratory diseases per 10μg/m^3^ increase in concentrations of PM_2.5_ stratified by genders. All the models were adjusted with public holidays, DOW and calendar day.


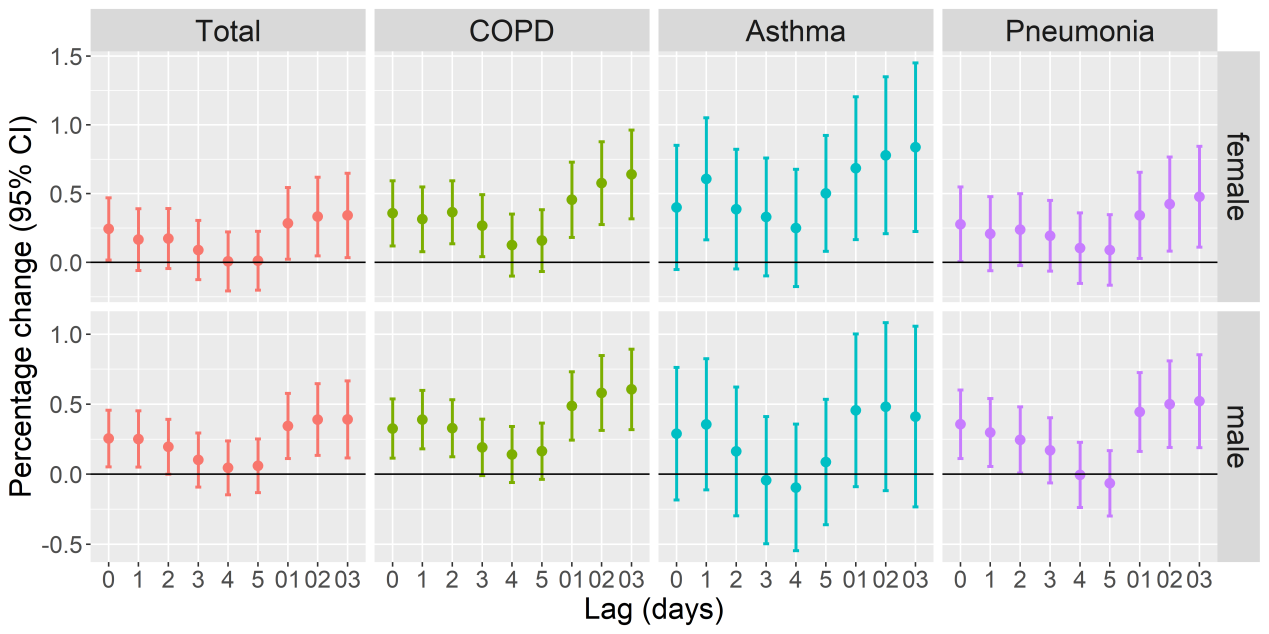


Fig. S II Percentage change with 95% confidence interval in hospital admissions for respiratory diseases per 10μg/m^3^ increase in concentrations of PM_10_ stratified by genders. All the models were adjusted with public holidays, DOW and calendar day.


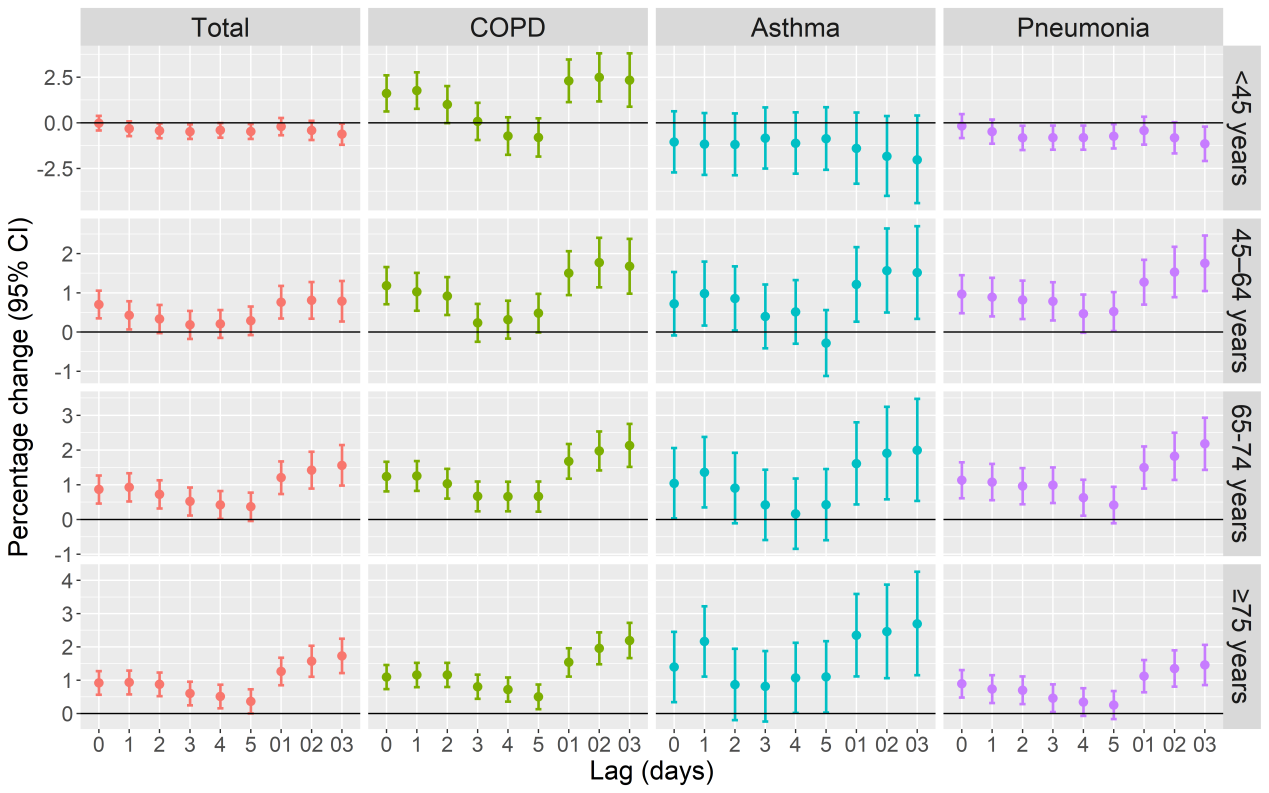


Fig. S III Percentage change with 95% confidence interval in hospital admissions for respiratory diseases per 10μg/m^3^ increase in concentrations of PM_2.5_ stratified by age groups. All the models were adjusted with public holidays, DOW and calendar day.


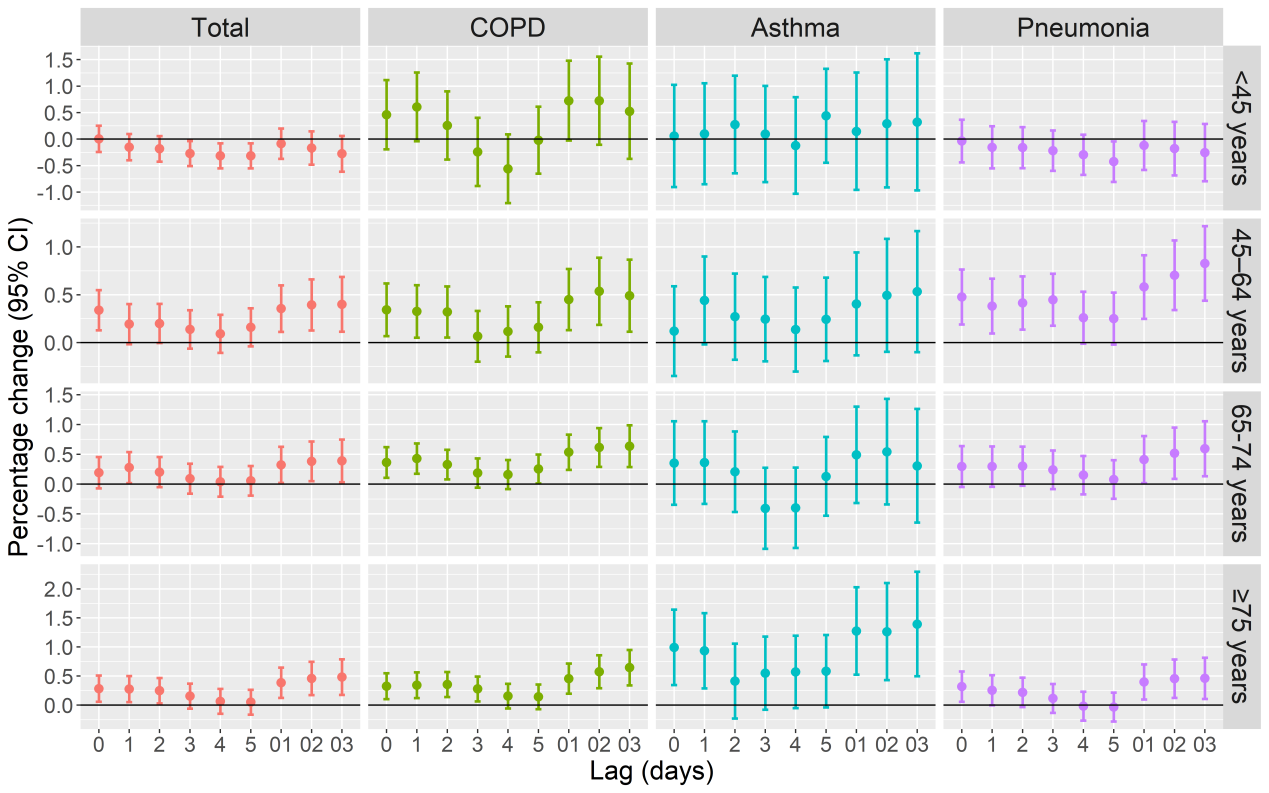


Fig. S IV Percentage change with 95% confidence interval in hospital admissions for respiratory diseases per 10μg/m^3^ increase in concentrations of PM_10_ stratified by age groups. All the models were adjusted with public holidays, DOW and calendar day.


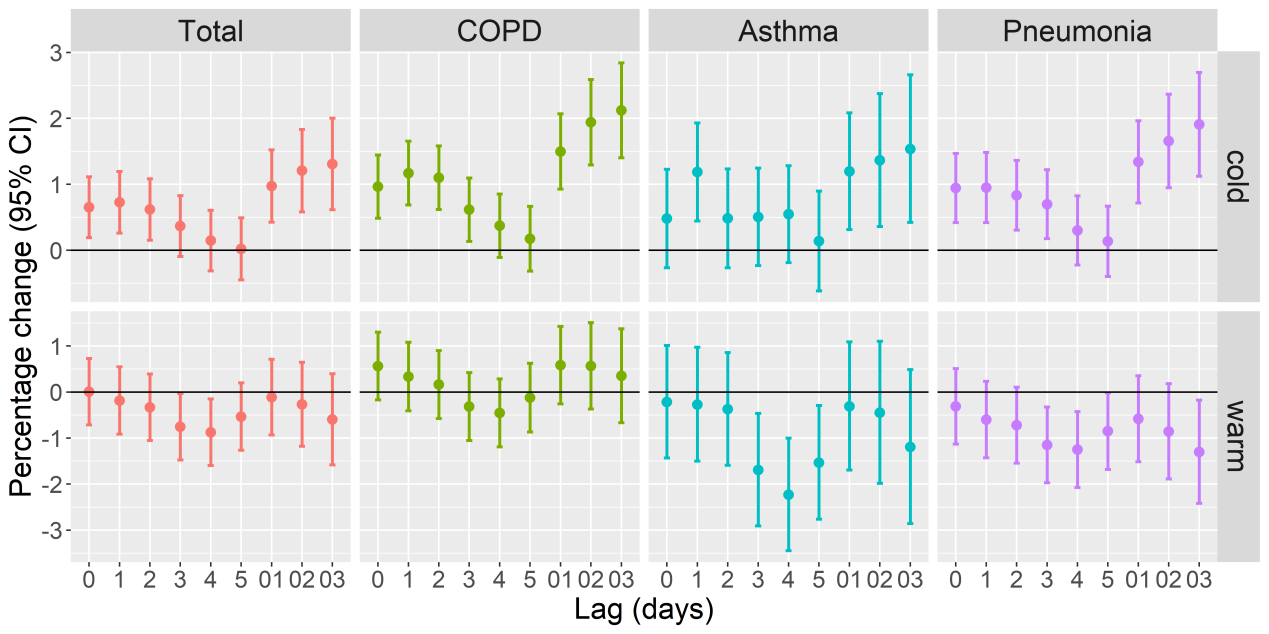


Fig. S V Percentage change with 95% confidence interval in hospital admissions for respiratory diseases per 10μg/m^3^ increase in concentrations of PM_2.5_ stratified by season. All the models were adjusted with public holidays, DOW and calendar day.


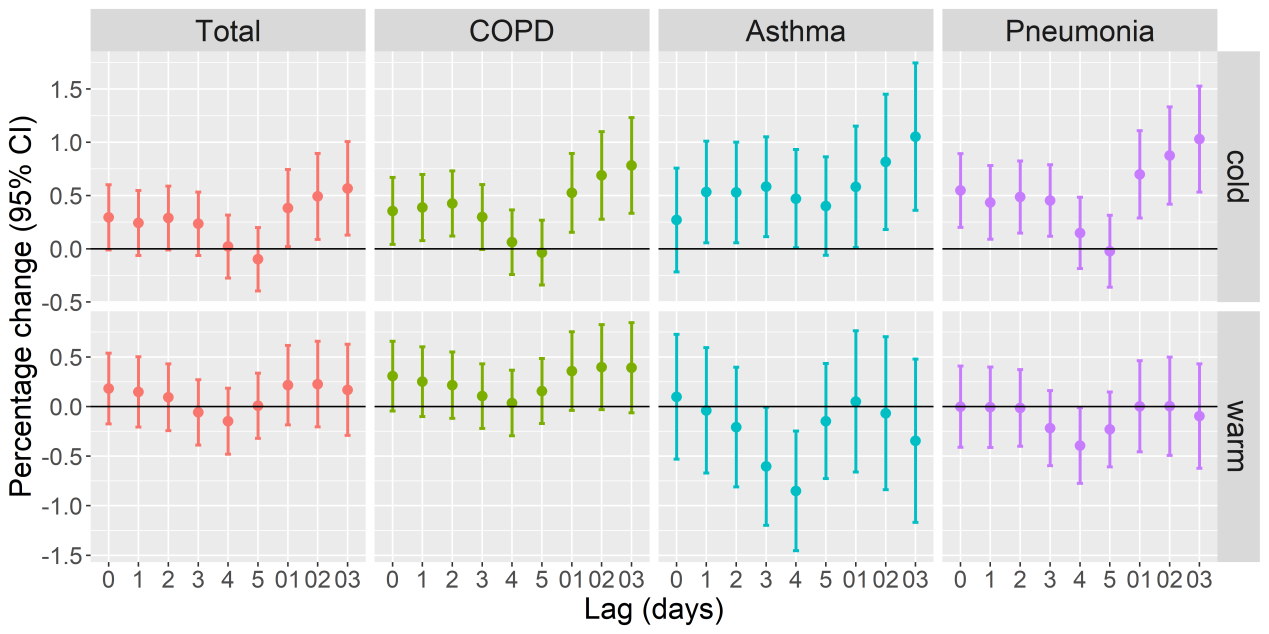


Fig. S VI Percentage change with 95% confidence interval in hospital admissions for respiratory diseases per 10μg/m^3^ increase in concentrations of PM_10_ stratified by season. All the models were adjusted with public holidays, DOW and calendar day.
